# Association of Circulating Serum miR-34a and miR-122 with Dyslipidemia among Patients with Non-Alcoholic Fatty Liver Disease

**DOI:** 10.1371/journal.pone.0153497

**Published:** 2016-04-14

**Authors:** Noel C. Salvoza, David C. Klinzing, Juliet Gopez-Cervantes, Michael O. Baclig

**Affiliations:** 1 Research and Biotechnology, St. Luke’s Medical Center, 279 E. Rodriguez Sr. Blvd., 1112 Quezon City, Philippines; 2 Liver Disease and Transplant Center, St. Luke’s Medical Center, 279 E. Rodriguez Sr. Blvd., 1112 Quezon City, Philippines; University College London, UNITED KINGDOM

## Abstract

Non-alcoholic fatty liver disease (NAFLD) covers a spectrum of diseases from simple steatosis to non-alcoholic steatohepatitis, with approximately 20% risk of progressing to fibrosis and cirrhosis. The aim of this study was to compare the relative expression levels of circulating miR-21, miR-34a, miR-122, miR-125b and miR-375 between healthy controls and NAFLD patients, and to assess the feasibility of microRNAs as potential biomarkers for NAFLD. A cross-sectional study was conducted to evaluate circulating serum miRNAs as potential diagnostic markers for NAFLD. Twenty-eight clinically diagnosed and histologically-confirmed NAFLD patients, as well as 36 healthy controls were enrolled in this study. The relative expression of serum microRNAs were calculated using the comparative cycle threshold with spiked-in *C*. *elegans* miR-39 as exogenous internal control. Serum levels of miR-34a and miR-122 were significantly higher in NAFLD patients than in healthy controls (*P* = <0.0001). Positive correlations were observed between serum miR-34a with very low density lipoprotein cholesterol (VLDL-C) and triglyceride levels. However, the expression levels of miR-34a and miR-122 did not correlate with the histological features of NAFLD. Interestingly, receiver operating characteristic (ROC) curve analysis revealed that miR-34a and miR-122 are potential markers for discriminating NAFLD patients from healthy controls with an area under the curve (AUC) values of 0.781 and 0.858, respectively. Serum levels of miR-34a and miR-122 were found to be significantly higher among NAFLD patients, and were positively correlated with VLDL-C and triglyceride levels. Thus, circulating miR-34a and miR-122 can be used as potential biomarkers for discriminating NAFLD patients from healthy controls. Larger cohorts are required to validate the utility of miR-34a and miR-122 in monitoring liver injury.

## Introduction

Non-alcoholic fatty liver disease (NAFLD) is characterized by excessive fat accumulation, as well as hepatic cellular degeneration without a history of excessive alcohol intake, and in the absence of other known liver diseases such as chronic hepatitis B (CHB) and chronic hepatitis C (CHC) virus infection. NAFLD can be categorized into 2 phenotypes: non-alcoholic fatty liver (NAFL) and non-alcoholic steatohepatits (NASH). NAFL is defined as the presence of liver steatosis without evidence of hepatic damage which is usually non-progressive, while NASH is often progressive and can lead to cirrhosis and hepatocellular carcinoma [1–2].

Recent studies estimate that 30% to 40% of adults in the United States are affected by NAFLD [3]. In Europe, the prevalence of NAFLD varied from 2% to 44% [4]. It has been reported that Hispanics bear the highest propensity to develop NAFLD with a prevalence of 45% [5]. In Asia, the prevalence of NAFLD was 15% to 45% [6]. It is estimated that 30% to 40% of patients with NASH may develop liver fibrosis; about 15% to 20% may develop cirrhosis and 2% to 3% may develop hepatocellular carcinoma. Children with NAFLD may later on develop end-stage liver disease and possibly need transplantation [3].

The principal risk factors associated with NAFLD include metabolic syndrome such as obesity, type-2 diabetes and hyperlipidemia. In particular, the prevalence of obesity in European patients with NAFLD varied from 25% to 94%. For type-2 diabetes, the prevalence ranged from 40% to 70%, while the prevalence of hyperlipidemia in patients with NAFLD varied from 20% to 92% [5,7]. Indeed, weight loss and exercise can play an important role in the management of NAFLD.

Ultrasound is the widely used imaging test for NAFLD and the most inexpensive diagnostic modality. However, this technique is not sensitive if 30% or less of the area is affected by liver steatosis. On the other hand, computerized tomography (CT scan) is accurate for diagnosing moderate-to-severe liver steatosis. However, CT scan is not accurate for detecting mild steatosis [3,8]. Magnetic resonance imaging (MRI) has demonstrated good sensitivity, as well as specificity in detecting liver steatosis. However, MRI is not widely available and is costly. Meanwhile, transient elastography has been reported to be unreliable in approximately 15% of NAFLD patients due to obesity [9–10]. Controlled attenuation parameter (Fibroscan^®^) provides a high accuracy to identify low steatosis compared with ultrasound. However, this method is limited by body mass index (BMI) and requires further validation using larger cohorts [11–13]. It is widely accepted that liver biopsy is the gold standard for the diagnosis of NAFLD and is an effective tool for prognostication. However, this technique is invasive and occasionally associated with severe complications. Thus, the identification of diagnostic and prognostic markers for NAFLD is needed [14].

MicroRNAs are highly conserved non-coding RNAs, approximately 18–25 nucleotides long that regulate gene expression. They are stable in extreme conditions such as low or high pH, extreme temperature and RNAse activity [15–16]. MicroRNAs are present in almost all body fluids such as serum, plasma and urine. The profile and abundance of microRNAs have been correlated with various disease states including metabolic disease, chronic hepatitis B, chronic hepatitis C and cancer. It is well recognized that miR-122 is the most abundant miRNAs in hepatocytes accounting for approximately 70% of all hepatic microRNAs. Other candidate microRNAs such as miR-21, miR-34a, miR-122, miR-125b and miR-375 are known to help regulate inflammation, lipid and cholesterol metabolism in human or mouse liver tissues [17–20].

Several methods for gene expression of miRNA have been reported such as quantitative real-time PCR (qRT-PCR), next-generation sequencing (NGS) and microarray [19–24]. In this study, qRT-PCR was used to evaluate the relative expression of circulating serum miR-21, miR-34a, miR-122, miR-125b and miR-375 among healthy controls and NAFLD patients. A total of five circulating miRNAs (miR-21, miR-34a, miR-122, miR-125b, miR-375) were selected because they have been reported to be related to NAFLD pathogenesis, inflammation, lipid and cholesterol metabolism, fibrosis and hepatocellular carcinoma [18,20].

The threshold cycle (Ct) was normalized to synthetic spiked-in *C*. *elegans* miR-39. The serum miR-21, miR-34a, miR-122, miR-125b and miR-375 levels among NAFLD patients at various stages of liver damage such as steatosis, fibrosis and inflammation were also analyzed. In addition, the feasibility of serum miR-21, miR-34a, miR-122, miR-125b and miR-375 as potential biomarkers for NAFLD was assessed by ROC curve analysis. MicroRNA expression patterns associated with liver injury could provide useful information for developing novel biomarkers for NAFLD.

## Materials and Methods

### Serum Samples and Patient Groups

Serum samples were collected from 36 healthy controls and 28 biopsy-proven NAFLD patients. The healthy participants presented with normal BMI, normal ultrasound, normal blood chemistry such as serum fasting blood sugar (FBS), creatinine, blood urea nitrogen (BUN), uric acid, total cholesterol, triglycerides, high density lipoprotein cholesterol (HDL-C), low density lipoprotein cholesterol (LDL-C), very low density lipoprotein cholesterol (VLDL-C), alkaline phosphatase, total bilirubin, conjugated bilirubin, unconjugated bilirubin, total protein, albumin, globulin, albumin globulin ratio, aspartate transaminase (AST) and alanine transaminase (ALT). In addition, the control group was found to be negative for viral hepatitis A, B and C. In this study, liver steatosis was determined non-invasively by ultrasound (Acuson S2000, Siemens Medical Solutions, Mountain View, California, USA) and operated by an experienced radiology technician. However, liver biopsy was not done among healthy controls due to ethical reasons. For NAFLD patients, the inclusion criteria were as follows: liver steatosis in at least 5% of hepatocytes, less than 25 ml/day of alcohol intake and BMI of 18.5 kg/m^2^ to 25 kg/m^2^ [25–27]. Patients with evidence of autoimmune disease, CHB and CHC were excluded. All participants gave written informed consent prior to enrollment upon referral to the Liver Disease and Transplant Center. The study was approved by the St. Luke’s Institutional Ethics Review Committee. The sample population was computed using the OpenEpi v. 2.3.1 and G power with confidence interval of 95% and power of 95%.

### RNA Extraction, cDNA Synthesis and qRT-PCR

Total RNA was extracted from serum by Trizol^®^ LS Reagent (Life Technologies). Synthetic spiked-in *C*. *elegans* miR-39 was added to the serum prior to RNA extraction as internal control. The concentration and quality of the RNA was measured by Quantifluor^™^ RNA Dye System. Reverse transcription was done using TaqMan microRNA reverse transcription kit (Life Technologies). Expression of mature miRNA was detected using the TaqMan microRNA assay (Applied Biosystems). Real-time PCR was performed on the Rotor-Gene Q instrument (Qiagen, Hilden, Germany) using the following conditions: 95°C for 10 min, followed by 50–60 cycles at 95°C for 15 sec and 60°C for 60 sec. The relative expression levels of serum miR-21, miR-34a, miR-122, miR-125b and miR-375 were calculated using the comparative Ct values.

### Liver Histology

The histological status of liver biopsies obtained in 28 NAFLD patients was assessed using the Metavir Scoring System and Knodell Histology Index.

### Statistical Analysis

Patients’ clinical data were presented as mean ± SD. The data were log-transformed for analysis due to the magnitude and range of relative microRNA expression levels observed. Wilcoxon-Mann-Whitney test was used to evaluate expression differences of microRNAs between NAFLD patients and healthy controls. Kruskal-Wallis test with Bonferroni correction post-test was used to determine the differences of serum miR-21, miR-34a, miR-122, miR-125b and miR-375 levels in different disease states. A probability level of *P* = <0.05 was considered statistically significant and all tests were two-sided. The correlation coefficients r was calculated by using the Spearman correlation. ROC curve was constructed and the area under the curve (AUC) was calculated to evaluate the sensitivity and specificity of serum microRNA levels for discriminating healthy controls and NAFLD patients. Youden index was used for identification of the optimal cut-off point. Data were as analyzed using the SPSS 20.0 software.

## Results

Table 1 shows the demographic and clinical characteristics of healthy controls and NAFLD patients. There is a significant difference in age distribution (*P* = <0.001) and BMI (*P* = <0.001) but not in sex between the two groups. The serum FBS, triglyceride, HDL-C, VLDL-C, AST and ALT levels were found to be significantly different between heathy controls and NAFLD patients (*P* = <0.001). Positive correlations were observed between serum miR-34a with VLDL-C (r = 0.44) and triglyceride (r = 0.43) levels.

**Table 1:** Demographic and clinical characteristics of healthy controls and NAFLD patients.

Parameters	Healthy controls	NAFLD patients	*P* value
	N = 36	N = 28	
	N (%)	N (%)	
Sex			
Male	22 (61)	13 (46)	NS
Female	14 (39)	15 (54)	NS
	**Mean ± SD**	**Mean ± SD**	
Age	33.2 ± 10.3	51.6 ± 13.1	0.001
BMI (kg/m^2^)	22.1 ± 1.8	28.5 ± 5.9	0.001
FBS (mg/dl)	88.7 ± 8.3	112.3 ± 31.6	0.001
Creatinine (mg/dl)	1.0 ± 8.3	1.0 ± 0.2	NS
BUN (mg/dl)	11.4 ± 3.1	13.1 ± 4.1	NS
Uric acid (mg/dl)	5.5 ± 1.5	6.0 ± 1.3	NS
Cholesterol (mg/dl)	198 ± 37.8	206.2 ± 49.0	NS
Triglycerides (mg/dl)	71.5 ± 46.2	153.4 ± 71.7	0.001
HDL-C (mg/dl)	59.9 ± 16.4	45.9 ± 14.1	0.001
LDL-C (mg/dl)	122.1 ± 31.5	122.1 ± 39.7	NS
VLDL-C (mg/dl)	14.4 ± 9.3	30.7 ± 14.4	0.001
Non-HDL-C (mg/dl)	139.0 ± 37.2	160.4 ± 47.4	0.048
AK (IU/L)	96.9 ± 20.0	95.7 ± 40.2	NS
TB (mg/dl)	0.8 ± 0.4	0.8 ± 0.9	NS
CB (mg/dl)	0.1 ± 0.1	0.2 ± 0.6	NS
UB (mg/dl)	0.7 ± 0.3	0.6 ± 0.4	NS
Total protein (g/dl)	7.9 ± 0.4	8.2 ± 0.6	NS
Albumin (g/dl)	4.3 ± 0.2	3.8 ± 0.3	NS
Globulin (g/dl)	3.6 ± 0.4	4.3 ± 0.5	NS
A/G ratio	1.2 ± 0.1	0.9 ± 0.1	NS
AST (IU/L)	21.3 ± 7.6	45.9 ± 26.2	0.001
ALT (IU/L)	42.1 ± 14.8	85.8 ± 46.1	0.001

Independent t-test was performed to determine the differences between the two groups. Abbreviation: BMI = body mass index, FBS = fasting blood sugar, BUN = blood urea nitrogen, HDL-C = high density lipoprotein cholesterol, LDL-C = low density lipoprotein cholesterol, VLDL-C = very low density lipoprotein cholesterol, Non-HDL-C = non-high density lipoprotein cholesterol, AK = alkaline phosphatase, TB = total bilirubin, CB = conjugated bilirubin, UB = unconjugated bilirubin, A/G ratio = albumin globulin ratio, AST = aspartate transaminase, ALT = alanine transaminase, NS = not significant

**Fig 1:**
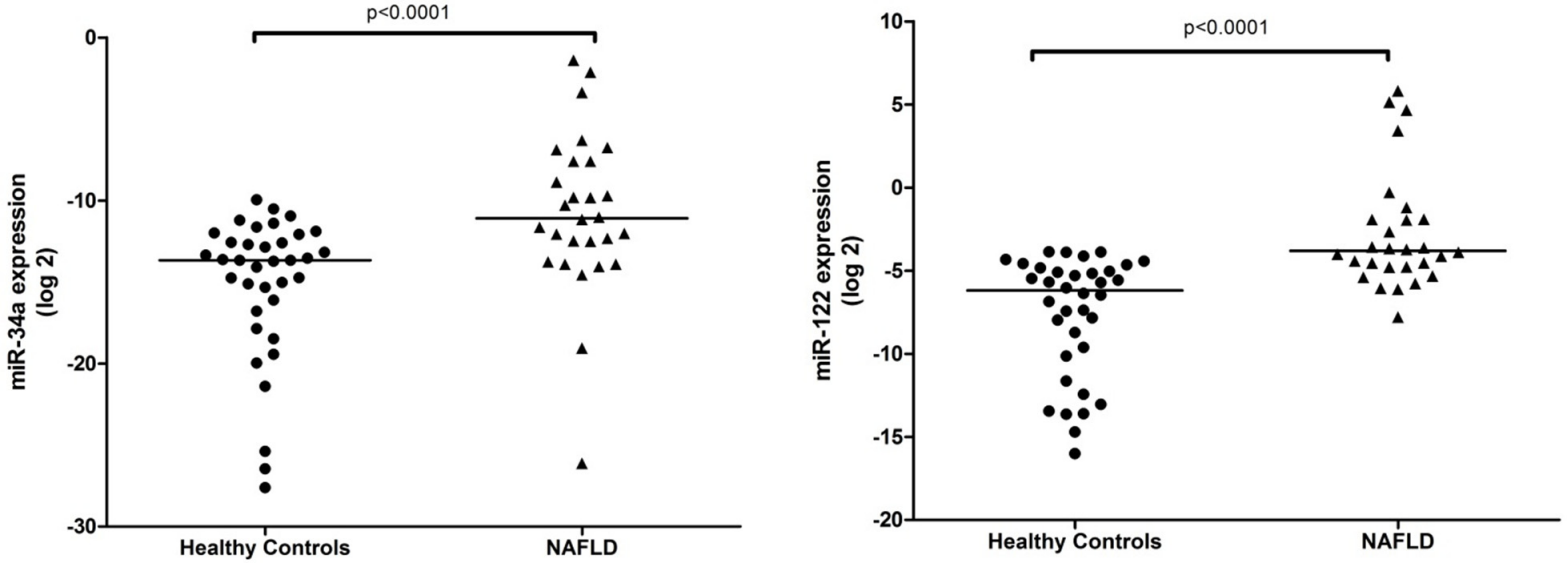
Up-regulation of serum miR-34a and miR-122 in NAFLD patients. Serum expression levels of miR-34a and miR-122 were measured in healthy controls and NAFLD patients. The relative expression levels were normalized to synthetic spike-in *C*. *elegans* miR-39. The *P* value was calculated according to Mann-Whitney U test. The horizontal lines indicate the medians.

The serum levels of miR-34a and miR-122 were significantly different between healthy controls and NAFLD patients (Fig 1). No significant differences were found in the expression levels of serum of miR-21, miR-125b and miR-375 between the two groups (data not shown).

**Fig 2:**
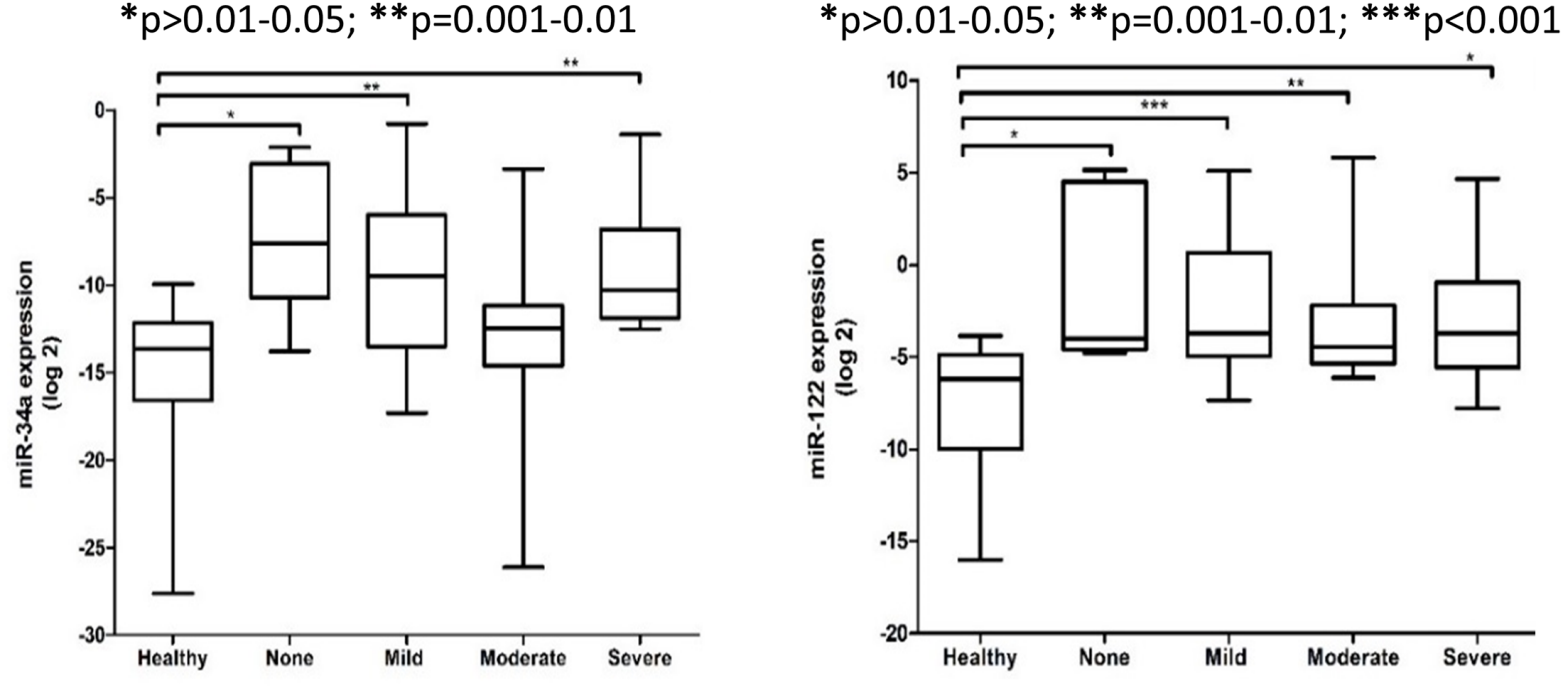
Relative expression of serum miR-34a and miR-122 among healthy controls and NAFLD patients at various degree of liver steatosis. The *P* values were calculated according to Kruskal-Wallis test with Dunn’s multiple comparison post-test. In the boxplots, the vertical lines indicate the range and the horizontal boundaries of the boxes represent the first and third quartile. The lines inside the boxes denote the medians. * p>0.01–0.05; ** p = 0.001–0.01; *** p<0.001.

**Fig 3:**
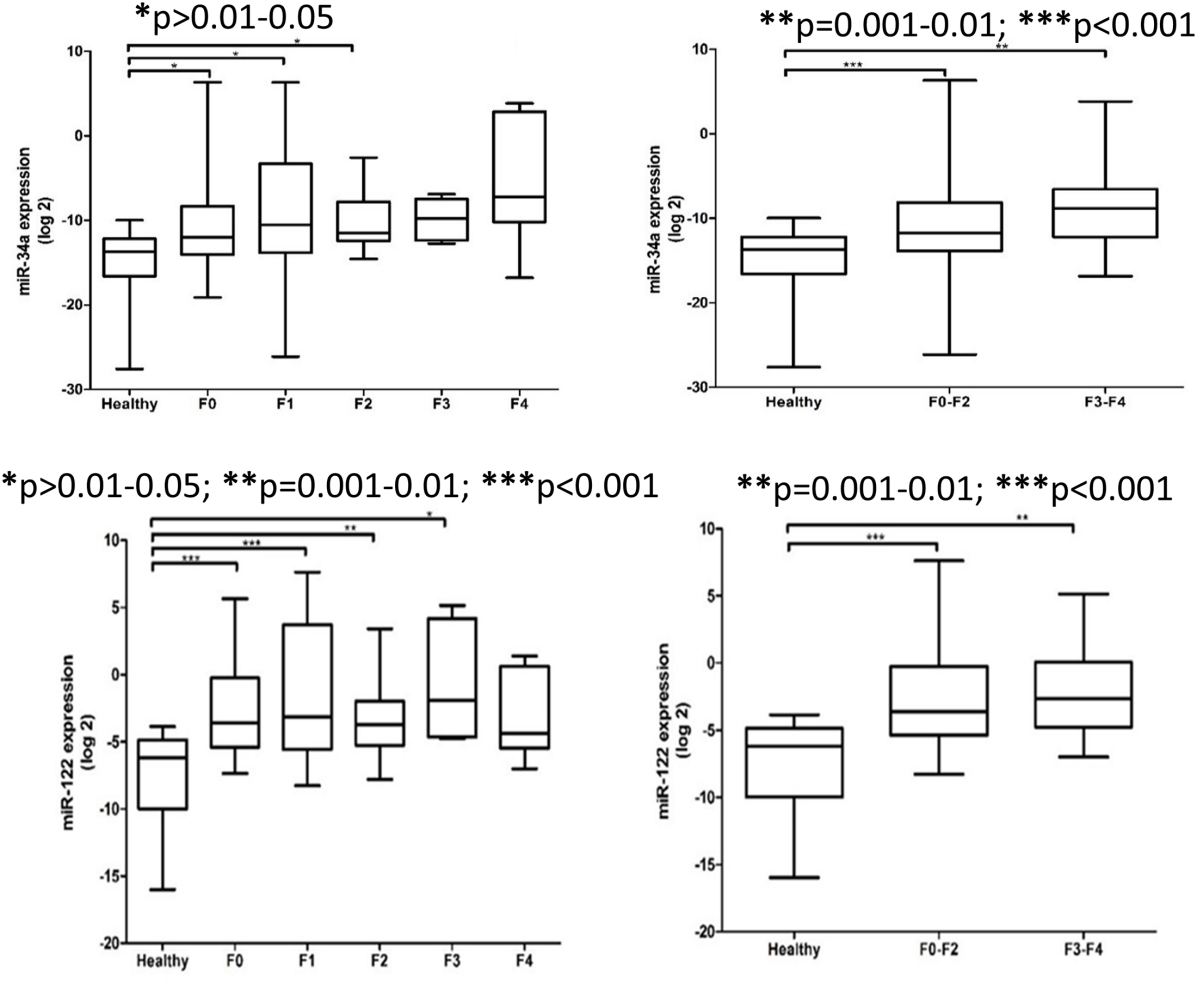
Relative expression of serum miR-34a and miR-122 among healthy controls and NAFLD patients according to stage of fibrosis and combined stages of fibrosis. The *P* values were calculated according to Kruskal-Wallis test with Dunn’s multiple comparison post-test. In the boxplots, the vertical lines indicate the range and the horizontal boundaries of the boxes represent the first and third quartile. The lines inside the boxes denote the medians. * p>0.01–0.05; ** p = 0.001–0.01; *** p<0.001.

**Fig 4:**
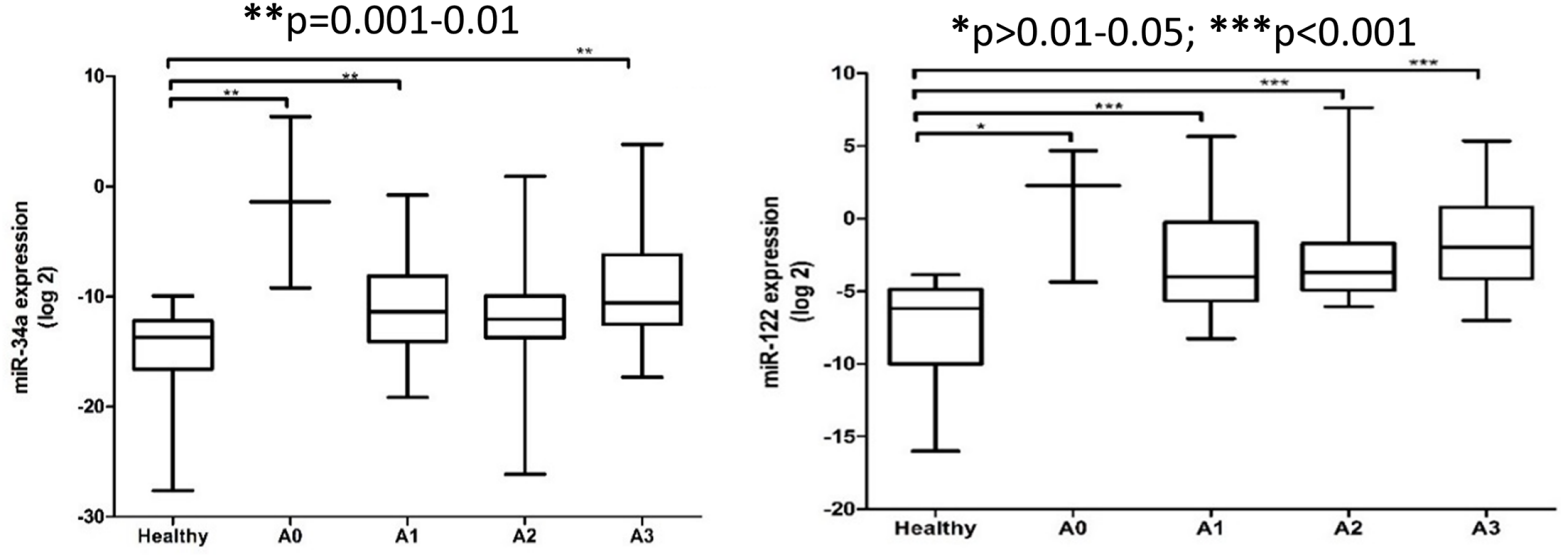
Relative expression of serum miR-34a and miR-122 among healthy controls and NAFLD patients at various degree of inflammation. The *P* values were calculated according to Kruskal-Wallis test with Dunn’s multiple comparison post-test. In the boxplots, the vertical lines indicate the range and the horizontal boundaries of the boxes represent the first and third quartile. The lines inside the boxes denote the medians. * p>0.01–0.05; ** p = 0.001–0.01; *** p<0.001.

In this study, the expression levels of serum miR-34a and miR-122 patients were significantly different between healthy controls and NAFLD patients based on the severity of liver steatosis, fibrosis and inflammation (Figs 2–4). The relative expression levels of serum miR-34a and miR-122 and clinical features of NAFLD patients are shown in Table 2. Although the expression levels of serum miR-122 tended to increase with increasing severity of liver injury, the observations did not reach statistical significance.

**Table 2:** Relative expression levels of serum miR-34a, miR122 and histological features of NAFLD patients.

Parameters	N	%	miR-34a	miR-122	*P* value
			mean ± SD	mean ± SD	
**Sex**					NS
Male	13	46.4	0.03 ± 0.07	0.09 ± 2.93	
Female	15	53.6	0.03 ± 0.10	7.85 ± 17.2	
**Steatosis**					NS
None	5	17.9	0.05 ± 0.10	9.22 ± 15.3	
Mild	4	14.3	0.01 ± 0.01	0.16 ± 0.12	
Moderate	11	39.3	0.01 ± 0.03	5.24 ± 16.9	
Severe	8	28.6	0.05 ± 0.13	3.29 ± 8.89	
**Fibrosis**					NS
F0	13	46.4	0.06 ± 0.12	2.03 ± 6.98	
F1	4	14.3	0.01 ± 0.01	14.2 ± 28.2	
F2	8	28.6	0.01 ± 0.01	1.51 ± 3.72	
F3	3	10.7	0.01 ± 0.01	11.9 ± 20.3	
**Inflammation**					NS
A0	1	3.6	0.38	25.2	
A1	12	42.9	0.03 ± 0.07	3.02 ± 10.2	
A2	9	32.1	0.01 ± 0.01	7.50 ± 18.7	
A3	6	21.4	0.01 ± 0.01	0.27 ± 0.28	

Abbreviation: NS = not significant

**Fig 5:**
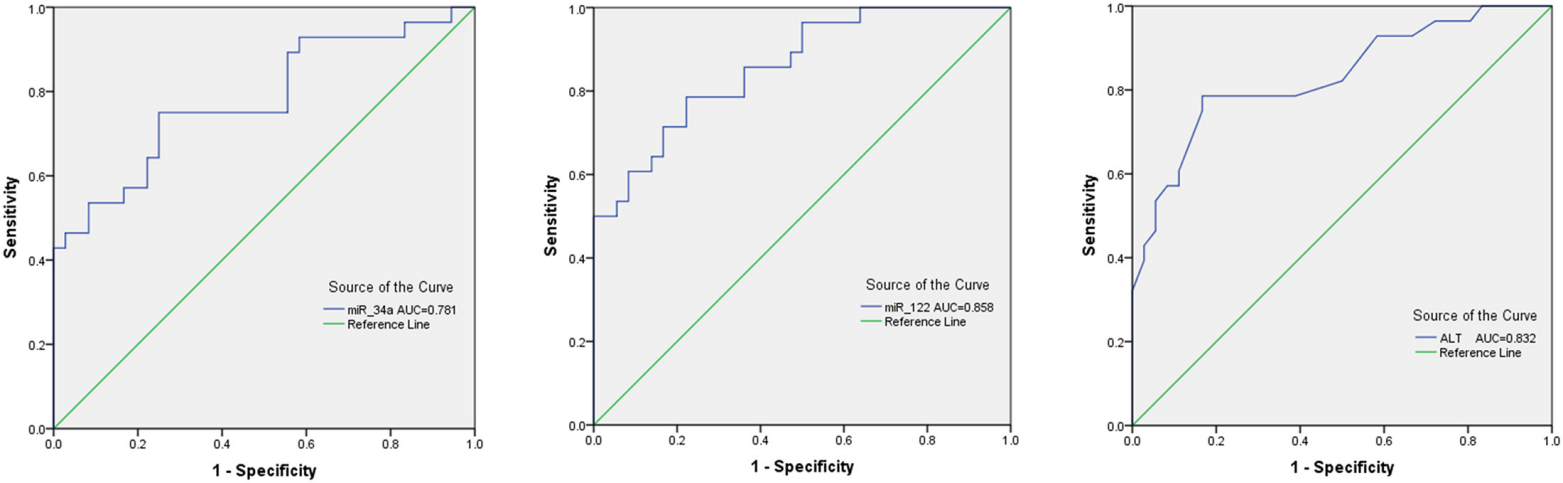
ROC curve analysis for serum miR-34a, miR-122 and ALT. The AUC for serum miR-34a, miR-122 and ALT are 0.781, 0.858 and 0.832, respectively.

The diagnostic values of serum miR-34a (AUC = 0.781; 95% CI = 0.663–0.899; *P* = 0.001) and miR-122 (AUC = 0.858; 95% CI = 0.769–0.947; *P* = 0.001) are comparable to serum ALT (AUC = 0.832; 95% CI = 0.729–0.935; *P* = 0.001) in discriminating NAFLD patients with healthy controls (Table 3 and Fig 5).

**Table 3:** Diagnostic performance of serum miR-34a, miR-122 and ALT in healthy controls and NAFLD patients.

Test	Sensitivity	Specificity	Youden's Index J COV	AUC	*P* value	95% CI
miR-34a	75.0%	75.0%	0.0002	0.781	0.001	0.663–0.899
miR-122	78.6%	77.8%	0.3570	0.858	0.001	0.769–0.947
ALT	78.6%	83.3%	50.50	0.832	0.001	0.729–0.935

Abbreviation: AUC = area under the curve, COV = cut-off value

## Discussion

The past decade has witnessed increasing interest in the discovery and development of non-invasive serum markers for NAFLD. Indeed, circulating microRNAs are potential biological markers because of their stability and presence in almost all body fluids [28–30]. Based on the results of this study, the serum levels of miR-34a and miR-122 were significantly different between healthy controls and NAFLD patients. This finding is in agreement with a previous study which showed that serum miR-34a and miR-122 reflects liver damage [20]. Cermelli and colleagues reported that miR-122 levels were increased by 7.2-fold among NAFLD patients compared to healthy controls. In addition, miR-34a levels were significantly increased to detectable levels among NAFLD patients but undetectable in plasma from healthy controls. More importantly, miR-122 and miR-34a levels correlated with steatosis grade, fibrosis stage and inflammation activity. In a cross-sectional study, Yamada et al. reported that serum level of miR-122 was correlated with the severity of liver steatosis. However, no significant difference was established between the expression level of serum miR-34a and disease severity [31]. In another study, the serum and hepatic miR-122 levels were found to be associated with hepatic steatosis and fibrosis [21]. From a clinical viewpoint, a specific miRNA signature or combined miRNA panel would be of interest in discriminating absent-to-mild fibrosis from moderate-to-severe fibrosis.

In this study, the relative expression levels of serum miR-34a and miR-122 were not correlated with the histological features of NAFLD. One of the possible reasons for this may be explained by the different normalization controls used in the study. In the present study, miR-39 was used for data normalization. Until now, there is no consensus on the use of housekeeping microRNAs for qRT-PCR [28,32]. Another possible reason may be due to variability in the sample collection and processing, RNA isolation and method of detection [22,33]. It has been suggested that a key prerequisite for adoption of microRNAs into clinical practice is standardization of protocol [18,34].

The feasibility of miR-122 as a marker for NAFLD was assessed by performing ROC curve analysis. In this study, the predictive value of serum miR-122 was higher than ALT. Results suggest that miR-122 may be used as a marker of liver injury and might have better sensitivity than liver enzyme test. This study corroborates with previous findings suggesting that serum level of miR-122 is suitable in diagnosing early onset of NAFLD and might be superior to serum ALT in assessing liver injury [35]. Clearly, liver enzymes are non-specific markers of liver injury and numerous studies have reported progressive NAFLD in spite of normal ALT levels.

Recently, it has been shown that non-HDL cholesterol (total cholesterol minus HDL-C) is an independent predictor for NAFLD [36]. Consistent with a previous report, non-HDL cholesterol was higher among NAFLD patients (160.4 ± 47.4) as compared with healthy controls (139.0 ± 37.2). Thus, the present study provided evidence that the non-HDL-C levels among NAFLD patients were statistically different from normal controls (*P* = 0.048). This finding is important to identify individuals at risk for developing hepatic steatosis and advanced liver injury so that prompt treatment can be initiated.

The regulation of lipid and cholesterol metabolism is mediated by a number of transcription factors such as liver X receptors (LXRs), sterol regulatory element-binding proteins (SREBPs), as well as non-coding RNAs. In particular, silencing of miR-122 can lead to an altered expression of lipid metabolism genes namely fatty acid synthase (FAS), 3- hydroxy-3-methylglutaryl-coenzyme A (HMG-CoA) reductase and SREBPs [19, 21,37].

Quantitative real-time PCR is the gold standard for measuring gene expression levels such as non-coding microRNAs. However, the presence of inhibitors in serum samples may limit the ability to extract RNA or the ability to accurately measure serum microRNAs by qRT-PCR [23,38]. In this study, synthetic, non-human (*C*. *elegans)* miR-39 was used as spiked-in control to ensure efficiency of RNA extraction.

Based on current literature, this study is the first to have reported the relative expression levels of serum miR-21, miR-34a, miR-122, miR-125b and miR-375 among Filipino patients with dyslipidemia and NAFLD. However, there are some limitations to this study. First, the cross-sectional research design will not allow correlation with disease progression to be drawn. Thus, a cohort study is needed to determine the possible role of serum miR-34a and miR-122 in the progression of NAFLD. Second, liver biopsies were not performed among healthy controls. Third, controlled attenuation parameter (Fibroscan^®^) and magnetic resonance spectroscopy were not done in this study. Fourth, although the detection of miRNAs require blood extraction and might cause minimal discomfort, it may serve as a complementary method to Fibroscan especially for obese individuals. Fifth, although comparable with a previous report [20], the sample size is small which may limit the ability to detect significant differences in the relative expression of serum miR-34a and miR-122 and the histological features of NAFLD. Thus, the authors consider this work as a pilot study and recommend that validation in an independent larger population is needed to confirm the findings.

In conclusion, serum levels of miR-34a and miR-122 were found to be significantly higher among NAFLD patients, and were positively correlated with VLDL-C and triglyceride levels. Thus, serum miR-34a and miR-122 can be used as potential biomarkers for discriminating NAFLD patients from healthy controls. Larger cohorts are required to validate the utility of serum miR-34a and miR-122 in monitoring liver injury.

## Supporting Information

S1 File Relative expression levels of serum miR-21, miR-125b and histological features of NAFLD patients. (DOC)

S2 File Diagnostic performance of serum miR-21 and miR-125b in healthy controls and NAFLD patients. (DOC)
