# Supplementary material for: Association of Circulating Serum miR-34a and miR-122 with Dyslipidemia among Patients with Non-Alcoholic Fatty Liver Disease
Source: PLoS One. 2016 Apr 14;11(4):e0153497. doi: 10.1371/journal.pone.0153497 (PMC4831793; doi:10.1371/journal.pone.0153497)
Supplement: S1 File — (DOC) [file pone.0153497.s001.doc]

**S1 File**. Relative expression levels of serum miR-21, miR-125b and histological features of NAFLD patients.

|  |  |  |  |  |  |
| --- | --- | --- | --- | --- | --- |
| **Parameters** | **N** | **%** | **miR-21** | **miR-125b** | ***P* value** |
|  |  |  | **mean ± SD** | **mean ± SD** |  |
| **Sex** |  |  |  |  | NS |
| Male | 13 | 46.4 | 0.02 ± 0.03 | 0.01 ± 0.07 |  |
| Female | 15 | 53.6 | 1.97 ± 5.73 | 0.07 ± 0.16 |  |
|  |  |  |  |  |  |
| **Steatosis** |  |  |  |  | NS |
| None | 5 | 17.9 | 0.28 ± 0.61 | 0.44 ± 0.96 |  |
| Mild | 4 | 14.3 | 0.01 ± 0.01 | 0.01 ± 0.01 |  |
| Moderate | 11 | 39.3 | 0.54 ± 1.71 | 0.04 ± 0.13 |  |
| Severe | 8 | 28.6 | 2.80 ± 7.76 | 0.06 ± 0.16 |  |
|  |  |  |  |  |  |
| **Fibrosis** |  |  |  |  | NS |
| F0 | 13 | 46.4 | 1.72 ± 6.09 | 0.04 ± 0.13 |  |
| F1 | 4 | 14.3 | 1.44 ± 2.84 | 0.11 ± 0.21 |  |
| F2 | 8 | 28.6 | 0.02 ± 0.04 | 0.01 ± 0.01 |  |
| F3 | 3 | 10.7 | 0.46 ± 0.78 | 0.07 ± 0.12 |  |
|  |  |  |  |  |  |
| **Inflammation** |  |  |  |  | NS |
| A0 | 1 | 3.6 | 22.0 | 0.46 |  |
| A1 | 12 | 42.9 | 0.12 ± 0.39 | 0.02 ± 0.06 |  |
| A2 | 9 | 32.1 | 0.67 ± 1.89 | 0.05 ± 0.15 |  |
| A3 | 6 | 21.4 | 0.04 ± 0.04 | 0.01 ± 0.01 |  |
|  | | |  |  |  |

| Abbreviation: NS = not significant | | | | | | |
| --- | --- | --- | --- | --- | --- | --- |
|  |  |  |  |  |  |  |
|  |  |  |  |  |  |  |
