# Supplementary material for: Association of Circulating Serum miR-34a and miR-122 with Dyslipidemia among Patients with Non-Alcoholic Fatty Liver Disease
Source: PLoS One. 2016 Apr 14;11(4):e0153497. doi: 10.1371/journal.pone.0153497 (PMC4831793; doi:10.1371/journal.pone.0153497)
Supplement: S2 File — (DOC) [file pone.0153497.s002.doc]

**S2 File.** Diagnostic performance of serum miR-21 and miR-125b in healthy controls and NAFLD patients.

| **Test** | **Sensitivity** | **Specificity** | **Youden's Index J COV** | **AUC** | ***P* value** | **95% CI** |
| --- | --- | --- | --- | --- | --- | --- |
| miR-21 | 53.6% | 80.6% | 0.0082 | 0.697 | 0.007 | 0.564-0.829 |
| miR-125b | 42.9% | 88.9% | 0.0007 | 0.661 | 0.028 | 0.523-0.799 |
|  | | | |  |  |  |

Abbreviation: AUC = area under the curve, COV = cut-off value
